# Retraction: Surface modified composite nanofibers for the removal of indigo carmine dye from polluted water

**DOI:** 10.1039/d5ra90104a

**Published:** 2025-09-12

**Authors:** M. G. Yazdi, M. Ivanic, Alaa Mohamed, A. Uheida

**Affiliations:** a Department of Applied Physics, KTH Royal Institute of Technology Stockholm Sweden alakha@kth.se salam@kth.se +201069676710 +46-8-7909132; b Division for Marine and Environmental Research, Ruder Boskovic Institute Zagreb Croatia; c Egypt Nanotechnology Centre, EGNC, Cairo University 6th October City 12588 Egypt; d Membrane Technology Department, Institute of Functional Interfaces (IFG), Karlsruhe Institute of Technology (KIT) 76344 Germany; e Production Engineering and Printing Technology Department, Akhbar El Yom Academy 12655 Giza Egypt

## Abstract

Retraction of ‘Surface modified composite nanofibers for the removal of indigo carmine dye from polluted water’ by M. G. Yazdi *et al.*, *RSC Adv.*, 2018, **8**, 24588–24598, https://doi.org/10.1039/C8RA02463D.

The Royal Society of Chemistry hereby wholly retracts this *RSC Advances* article due to concerns with the reliability of the data.

The SEM image of APAN nanofibers in Fig. 3a appears in multiple publications from this research group,^1–4^ and the authors have not been able to satisfactorily explain this.

Given the significance of these concerns, the Editor has lost confidence that the findings presented in this paper are reliable.

The authors were informed but have not indicated whether they agree with the decision to retract.

Signed: Laura Fisher, Executive Editor, *RSC Advances*

Date: 4th September 2025
